# Silver nanoparticle effect on *Salmonella enterica* isolated from Northern West Egypt food, poultry, and calves

**DOI:** 10.1007/s00253-022-12102-x

**Published:** 2022-08-10

**Authors:** Helmy Ahmed Torky, Samy Abd-Elsalam Khaliel, Eman Khalifa Sedeek, Rasha Gomaa Tawfik, Ahmad Abo Elmagd Bkheet, Sawsan Khamees Ebied, Heba said Amin, Samir Ibrahim Zahran, Hadeer Abd-Elhady Emara, Abeer Mohamad Nofal, Eman Moneer Elghazaly

**Affiliations:** 1 Department of Microbiology, Faculty of Veterinary Medicine, Alexandria University, Alexandria, Egypt; 2 Department of Microbiology, Faculty of Veterinary Medicine, Matrouh University, Matrouh, Egypt; 3 Department of Bacteriology, Damanhour Provincial Laboratory, Animal Health Research Institute, Dokky, Giza, Egypt; 4 Department of Bacteriology, Animal Health Research Institute, Alexandria Provincial Laboratory, Dokky, Giza, Egypt

**Keywords:** *Salmonella enterica*, Antimicrobial resistance, Silver nanoparticles, ERIC-PCR, Northern West Egypt

## Abstract

**Abstract:**

A total no. of 65 *Salmonella enterica* isolates recovered from food samples, feces of diarrheic calves, poultry, and hospital patient in large five cities at Northern West Egypt were obtained from the Department of Microbiology, Faculty of Veterinary Medicine, Alexandria University, Alexandria, Egypt. The 65 *Salmonella enterica* isolates had the *inv*A gene were grouped into 11 *Salmonella enterica* serovars with dominance of *S.* Enteritidis and *S.* Kentucky serovars. Their resistance pattern were characterized by using 18 antibiotics from different classes. Approximately 80% of the isolates were multidrug resistant (MDR). Enterobacterial repetitive intergenic consequences polymerase chain reaction (ERIC-PCR) typing of 7 strains of *S.* Enteritidis showed 5 clusters with dissimilarity 25%. *S.* Enteritidis clusters in 2 main groups A and B. Group A have 2 human strain (HE2 and HE3) and one food origin (FE7) with a similarity 99%. Group B divided into B1 (FE2) and B2 (FE3) with a similarity ratio ≥ 93%, while ERIC-PCR analysis of 5 strains of *S.* Kentucky revealed 4 ERIC types, clustered in 2 main groups A and B with similarity 75%. We studied the effect of silver nanoparticles (Ag-NPs) on 10 antibiotic resistant strains of *S*. Enteritidis and *S.* Kentucky. The broth microdilution minimum inhibitory concentration (MIC) and minimum bactericidal concentration (MBC) were detected. Evaluation of the affection using scanning electron microscopy (SEM) and transmission electron microscopy (TEM) showed different ratios of Ag-NPs and microorganism as well as at different contact time ended finally with morphological alteration of the bacteria. We submitted new method in vivo to explore the activity of nanosilver in chicken.

**Key points:**

• *Importance of ERIC-PCR to determine the relatedness between Salmonella isolates.*

• *Effect of silver nanoparticles to confront the antibacterial resistance*.

• *Studying the effect of silver nanoparticles in vivo on infected chicken with Salmonella*.

## Introduction

There are over 2500 serovars of *Salmonella enterica* (Andrews-Polymenis et al. 2010). Any serovar is thought to be capable of generating varied degrees of intestinal disease in people (Forshell and Wierup 2006). The majority of them are broad host range pathogens that infect a wide range of hosts, with only a few being host specific (Saroj et al. 2009); some serotypes are only found in certain parts of the world (Brands 2006), infecting animals, poultry, and fish, and are the leading cause of foodborne illness in humans globally (ECDC 2013).

According to the WHO, since 1990, *Salmonella* Enteritidis has been considered the most common cause of gastroenteritis worldwide (Chaitram et al. 2003) and salmonellosis in bovine, ovine, and poultry (Suh and Song 2006; Firoozeh et al. 2012; Dutta et al. 2012; Guizelini et al. 2019).

Meanwhile, *Salmonella* Kentucky isolation has been increased during recent years (Saroj et al. 2009; Osman et al. 2010a, b; Abd El-Ghany et al. 2012; Barua et al. 2012; Zahran et al. 2020) and as food borne pathogen both isolated by Shah et al. (2017) Shivaning Karabasanavar et al. (2020), Amin et al. (2021), Gawish et al. (2021), and Adel et al. (2021).

We require accurate subtyping information of strains to identify potential sources of infection, trace cross contamination, and pinpoint particularly virulent strains for efficient epidemiological surveillance and management of *Salmonella* species (Tenover et al. 1997; MacCannell 2013).

Increasing MDR *S. enterica* to cephalosporin and fluoroquinolones as critically important recommended treatment option (Chen and Schluesener 2008) will lead to increased severity, morbidity, and mortality of salmonellosis in humans and subsequently the use of the last line antimicrobials, e.g., cephapems (WHO 2021).

The prevalence of MDR *S. enterica* in Egypt, detected from retail meat samples, was 69.8% in 2010 and 82.4% and 100% in 2020 (Adel et al. 2021; Awad et al. 2020).

Remarkably, Egypt was formerly thought to be the source of highly drug-resistant *S.* Kentucky sequence type 198 (ST198-CipR) in Europe (Hawkey et al. 2019; Coipan et al. 2020), which was recently isolated from broilers in Lebanon (El Hage et al. 2020).

Several molecular techniques for typing *Salmonellae* have been proposed; the enterobacterial repetitive intergenic consequences polymerase chain reaction (ERIC–PCR), a simple technique of random amplified polymorphic DNA, has been successfully applied in genotyping of microbial pathogens, including gene mapping, detection strain diversity, population analysis, epidemiology, and demonstration of phylogenetic and taxonomic relationship (Li et al. 2009), without prior knowledge of target genome sequences (Maslow and Mulligan 1996; Stefańska et al. 2008; Li et al. 2009), faster with highest discriminatory power (Guimarães Ade et al. 2011) is an economical (Ranjbar et al. 2014) and capable of amplifying tiny amount of microbial DNA sequence.

As a result of the developed resistance of variant *Salmonella* species to antibiotics, which has become a major public health concern (Silver et al. 2006; Akinyemi et al. 2011), traditional antibiotics are being replaced by new alternative technologies such as nanotechnology, which has a wide range of potential applications in human and veterinary medicine (Rudramurthy et al. 2016). Silver nanoparticles are a suitable alternative among metallic nanoparticles with antibacterial activity because, in addition to possessing a strong antibacterial profile, they are also reasonably affordable to produce (Lee et al. 2007; Pal et al. 2007; Zhang et al. 2008; EL-sherif and Ali 2020) When comparing the studied nanoparticles, those with very low levels of MIC and MBC should be a focus in study, with concentration treatment and genus taken into consideration.

This work aimed to clarify the *Salmonella enterica* serovars and the benefit of silver nanoparticles (Ag-NPs) in the fight against the MDR bacterial strains. In the Northern West Egypt, starting from identification, antibiotic susceptibility testing, and the ability of silver nanoparticles in their application alone, take the opportunity of chicken as can be employed as a lab animal and host in vitro and in vivo. A specific attention to know if its antibacterial efficacy affected by methods of synthesis, concentration, time, and *Salmonella* serovars treated.

## Material and Methods

### Isolation and identification of *Salmonella enterica* from collected food samples

The preparation of all samples culturing and isolation of *Salmonella* was done according to the ISO 6579 (ISO 2002 and 2017). For isolation of *Salmonella*, swabs taken from humans or days old broiler chicks were performed as recorded by FDA.

### Identification of presumed *Salmonella* spp. was carried out by morphological and cultural characteristics following standard microbiological methods (Washington winner et al. 2001; Quinn et al. 2013)

**Fig. 1:**
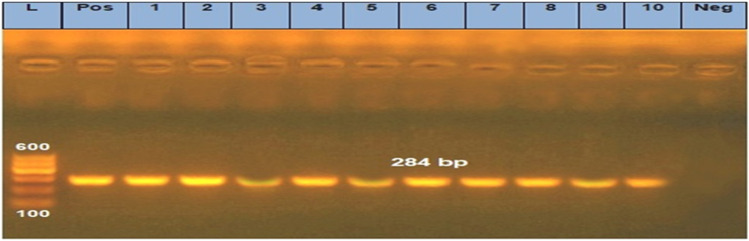
Agarose gel electrophoresis showing specific PCR using primer set for the invA gene. In this photo “Pos” stands for positive control, “Neg”; negative control and numbers indicate lanes with positive and negative isolates lane L (100–600 bp marker): all isolates (10) were positive that show specific band at 284 bp

Typical *Salmonella* morphology samples identified biochemically, were confirmed by PCR for the presence of invA gene as shown in Fig. 1, further serotyped using specific *Salmonella* O and H antisera (Difco, Franklien lakes, NJ, USA). All the serological identification and molecular characterization were performed at the Animal Health Research Institute, Dokki, Giza, Egypt.

#### Revival of bacterial strains

All bacteria isolated within 2020–2021 were revived in BHI broth (DIFICO) by overnight incubation at 37 °C, followed by plating on MacConkey’s agar (DIFICO) and confirmed serologically.

#### Antimicrobial susceptibility testing

For testing of antibiotic used, see Table 1; Kirby–Bauer disk diffusion assay was performed according to the standards and interpretive criteria described by clinical and laboratory standard institute (CLSI 2018).

**Table 1:** *Salmonella* serovar spp. and its phenotype resistance pattern

Serovar	Source	City	Number	Resistance phenotype pattern	Antibiotic disc (Oxoid)
*S.* Enteritidis	Cheese	Matrouh	1	GEN,N,SPT,CHL,AMP,CXM,CAZ,CTX	TET = Tetracycline AMP = B-lactam Ampicillin, AZ = Macrolides Azithromycin CH = chloramphenicol CTX = Cephalosporin (cefotaxime) SPM = Aminoglycoside (streptomycin) GEN = Aminoglycoside (gentamycin) LE = fluoroquinolone (levofloxacin) VAN = vancomycin, (glycopeptide) AMX = Amoxicillin AMP = Ampicillin pencillin/B lactam) IMP = Imipenem carbapenem N = Neomycin aminoglycoside CIP = Ciprofloxacin fluroquinolone CL = Colistin polymyxin CPM = cefipime 4thg cephalosporine NOR = norfloxacine = fluoroquinolone ATM = aztreonam = monobactam CAZ = ceftazidime = 3rdg cephalosporine CPD = cefpodoxime = 3rdg cephalosporin DO = doxycycline = tetracycline CPM = cefipime4thg cephalosporin SPT = spectinomycin = aminogcyclostol MOX = moxlfloxacin = Quinolones MEP = meropenem = carbapenem
	She camel milk		1	GEN,N,SPT,CHL,AMP,CXM,CAZ,CTX	
	Sheep meat		1	GEN,N,SPT,CHL,AMP,CXM,CAZ,CTX	
	Chicken meat	Alexandria	2	GEN,N,SPT,CHL,AMP,CXM,CAZ,CTX	
	Minced meat		1	GEN,AMK,CXM,AMP,TET	
	Chicken liver		1	GEN,AMK,CXM,AMP	
	Human stool		3	AMP,CXM,CIP,MOX,TET.IMP,MEP.GEN	
	Calf	Damnhour	1	CHL,N,AMX,N,CL,SPM,CIP	
	Calf		1	CHL,N,AMX,N,CL,SPM	
	poultry		1	GEN,VAN,SPI,AMP,NOR,IMP,AMX	
	Minced meat	Mahmoudia	5	AMK,NAN,SPM,GEN,NOR,IMP,TET	
	Giblets		3	CAZ,CTX,INP,MEP,GEN,AMK,CIP,TET	
			1	OXA,GEN,STR,TET,CTX,AMP,NAL	
	Chicken meat	Desouq	1	AMP,AMC,CHL,OXA,SPT,TET,NOR	
	Chicken meat		1	STR,OXA,TET,NAL,CTX	
S. Kentucky	Chicken meat	Matrouh	1	GEN,AMK,CEB,AMP,TET,CIP	
	Chicken meat	Alexandria	2	GEN,N,SPT,CXM,AMP,TET	
	Minced meat		1	AMP,CXM,CTX,AMP,TET	
	Human stool		2	GEN,AMK,AMP,TET,CIP	
	Minced meat	Damnhour	1	GEN,AMK,TET,CTX,AMP,TET	
	Giblets	Mahmoudia	1	GEN,AMC,SPM,AMK,VAN	
	Chicken nuggets		1	GEN,AMC,SPM,AMK, IPM	
	Chicken meat	Desouq	1	SPT,AMC,CTX,CHL	
	Chicken meat		1	SPT,AMC,LE,CTX,CIP,MOX,CPA	
	Chicken broilers		3	GEN,AMK,TET,CPA,MOX,TET	
S. Typhimurium	Chicken meat	Matrouh	1	AMC,STR,TET,GEN,CAZ,NAL,OXA	
	Chicken meat	Damnhour	1	AMC,AMP,ATM,TET,CTX	
	Diarrheic poultry		1	GEN,SPT,AMC,CHI,N,CL	
	Diarrheic calf		2	GEN,SPT,AMC,CHI,N,CL	
	Kariesh cheese	Mahmoudia	1	GEN,SPM,AMC,ANK,IPM,TET,CHL,STR	
	Cheese		1	OXA,STR,TET,AMP,GEN,CTX	
	Kariesh cheese		1	OXA,STR,TET,AMP,GEN,CTX	
	Burger		1	AMC,AMP,SPM,TET,CXM	
	Chicken meat	Desouq	1	AMC,AMP,ATM,GEN,SPM,TET	
	Chicken meat		1	AMP,ATM,SPM,TET,GEN	
	Beef meat		1	AMX,AMP,CHL,CTX,TET,GEN,SPM,CPD	
	Beef meat		1	CTX, GEN, AMK	
S. Infants	Chicken meat	Matrouh	1	AMC,AMP,GEN,AMK,CAZ,CIP,CTX,SPM	
	Calf	Damnhour	1	AMC,AMP,GEN,CHL,DO	
	Chicken meat	Desouq	1	TET, CHL, CTT, SPM, AMP, GEN, OXA	
	Beef meat		1	AMX, CTT, AMP, GEN, NAL	
S. Tennesee	Chicken meat	Alexandria	1	CHL, AMX, CXM, AMP, GEN	
S. London	5 days old chicken	Desouq	3	TET, CHL, SPM, AMP, GEN, AZ	
S. Ohio	Days old chicken	Desouq	1	TET, CHL, SPM, AMP, GEN	
S. Gallinarum	Poultry	Damnhour	1	GEV, N, AMX	
S. Newport	Poultry, calf	Damnhour	2	AMX, N	
S. Megherafell	Calf	Damnhour	1	CL, GEN, N	
S. Tsevi	Imported calf	Damnhour	1	GEN, N	

The reference strain, *Escherichia coli* ATCC 25,922, was included as quality control strains showed resistance to antibiotics from at least three different classes considered as MDR — multidrug resistant (Magiorakos et al., 2012). The identity of *S.* Enteritidis was confirmed by repetitive sequence PCR using primers described the resistant Strain previously (Suh and Song 2006).

For MDR *Salmonella* Kentucky to be used for Ag-NP effect investigation, all the serological identification and molecular characterization were performed at the Animal Health Research Institute, Dokki, Giza, Egypt.

#### ERIC-PCR

Primer pairs for ERIC-PCR amplification were as follows: ERIC IR (5-ATGAACTCCTGGGGATTCAC-3) and ERIC-2 (5-AAGTAAGTACTGGGGTGAGCG C-3) amplification and condition were performed as usual (Versalovic et al. 1991). The size of the amplified fragments was determined after electrophoresis in a submerged agarose gel (1.5%) (Sambrook et al. 1989).

#### Ag-NP synthesis methods’

The different Ag-NPs that varied in size, synthesis method, and properties are summarized in Table 2. Silver nitrate Ag No3; 99.9%, Sigma Aldrich, st, l0 Mo, USA was used.

**Table 2:** Bactericidal efficacy of Ag-NPs on *Salmonella enterica* serovar spp

Concentration	Size and shape	*S. enterica* serovar spp.	Method of preparation
MIC = 20 mg/ml MBC = 40 mg/ml	45 ± 5 nm round	*S. enterica* serovar Kentucky from days old chicks	Van Dong et al. (2012)
MIC = 10 mg/ml MBC = 20 mg/ml	45 ± 5 nm Round	*S. enterica* serovar London from days old chicks	Van Dong et al. (2012)
MIC = 25 ppm MBC = 50 ppm	26.5 nm Round	*S. enterica* serovar Enteritidis from food origin	Abdelsalam et al. (2019)
MIC = 25 ppm MBC = 50 ppm	30–40 nm Round	*S. enterica* serovar Enteritidis *S. enterica* seovar Kentucky from clinical isolates	Abdelsalam et al. (2019)

### Susceptibility of *Salmonella* spp. to Ag-NPs

The broth microdilution briefly minimum inhibitory concentration (MIC) and minimal bactericidal concentration (MBC) were conducted to measure the in vitro activity of Ag-NPs against each bacterial strain as conducted by (Wikler et al. 2008).

#### Ultra structure observations

Particle size and shape were determined by TEM (transmission electron microscope) and SEM (scanning electron microscope) utilization (Samberg et al. 2011); images were taken prior to post staining to show the location of Ag-NPs relative to bacteria and post stained with lead citrate, uranyl acetate in order to visualize cell morphology and membrane integrity.

##### In vivo evaluation of Ag-NP effect on S. Kentucky virulence

This in vivo evaluation Ag-NP effect of silver nanoparticles on the virulence of *S.* Kentucky was carried out by inoculating it in newly hatched days old broiler chicks from the Cobb lineage of breeding hens instead of SPF chicks. Days-old chicks were free from *Salmonella* infection (–v slide agglutination test and *Salmonella* isolation culture free).

The birds were kept in 32 °C heated environment and housed in isolated boxes with 30 cm length × 55 cm height × 35 cm breadth. Drinkable water and antibiotic free commercial food were provided.

The *S*. Kentucky was spread on XLD agar and incubated for 24 h at 37 °C. Three colonies were selected and removed to 10 ml of BHI broth and incubated for 24 h at 37 °C.

The chicks were randomized into three groups. Each group contains five chicks; the first group of chicks was infected by crop gavage with 0.5 ml of previously prepared broth culture containing 1 × 10^6^ colony forming units of the *S.* Kentucky (Dhillon et al. 2001).

The second group was infected with *S.* Kentucky and 20 mg/ml silver nanoparticles, and was observed for 1 week and record observation (Osman et al. 2010a). The third group was injected with silver nanoparticles only.

All broiler chicks were investigated daily from the second day after injection for detecting shedding of *Salmonella* in feces by culturing on XLD medium.

At the end of the week, the chicks were euthanized through cervical dislocation to detect the presence of *Salmonella* spp. from re-isolation and slide agglutination reaction with somatic (O) antisera from liver and spleen (Borsoi et al. 2009).

The macroscopic lesions were observed such as airsacculitis, peritonitis, perihepatitis, pericarditis, and cellulitis, in the dead or killed chicks.

The chicks that survived until the seventh day to tenth day were killed by cervical dislocation, necropsied, and evaluated as explained before.

## Results

A total of 65 *Salmonella enterica* strains included from 11 serovar species were revealed from different food samples including, chicken, beef, goat’s meat, hamburger, milk, cheese, and the feces of diarrheic calves and poultry. The investigation of 5 large cities in Northern West Egypt, Matrouh, Alexandria, Damnhour, Mahmoudia, and Desouq during the period time 2020–2021 ended by investigation of days old chick isolation of *Salmonellae* (*n* = 3 S. Kentucky, *n* = 3 S. London, one S. Ohio) at 2022 with the dominance of the serovar *Salmonella* Enteritidis and emergency of *S.* Kentucky among the most prevalence serotypes. All the types showed resistance to at least 3 of these antibiotics (ampicillin, oxacillin, streptomycin, and tetracyclin). Most of strains showed MDR phenotype (multidrug resistant) resist more than 3 classes of antibacterial as shown in Table 1.

**Fig. 2:**
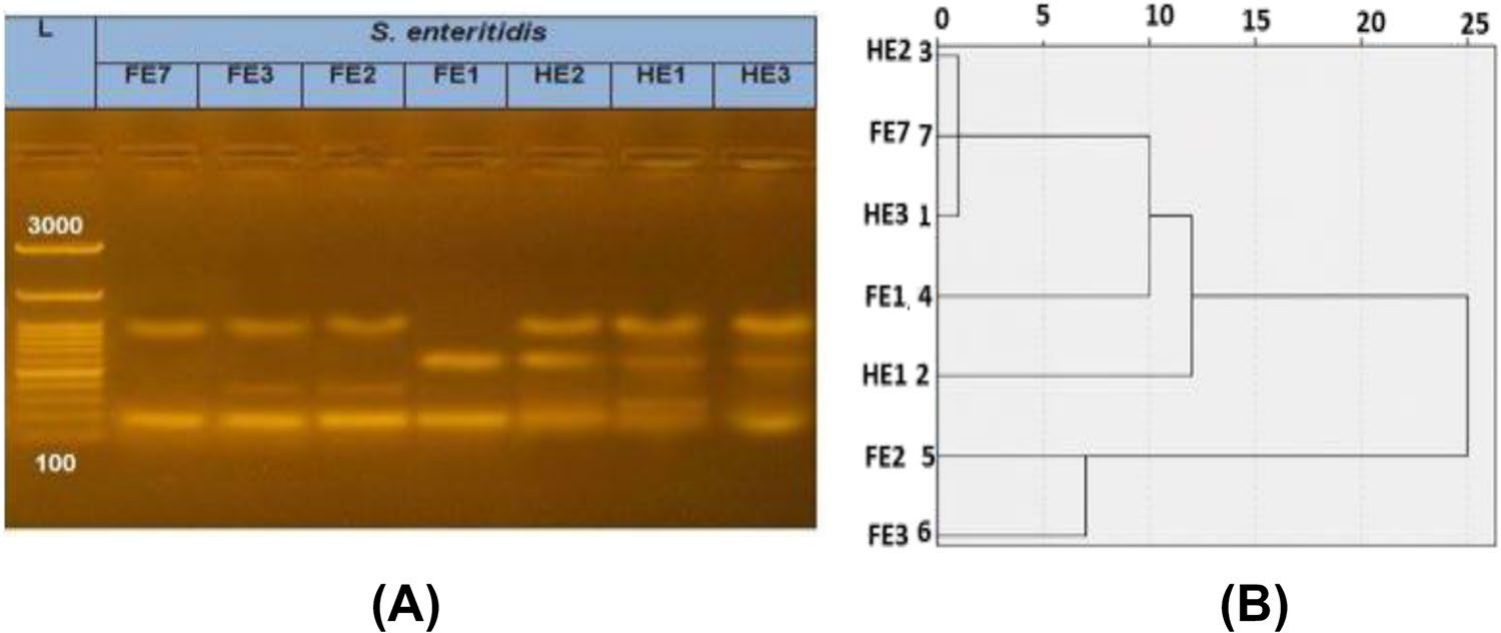
ERIC-PCR of *Salmonella* Enteritidis. **A**. ERIC-PCR finger printing of 7 *S.* Enteritidis isolates in 1.5% agarose gel, L: 100 bp molecular marker, HE1, HE2 and HE3: *S. *Enteritidis isolates from human origin, FE1, FE2, FE3 and FE7: *S.* Enteritidis isolates from food origin. **B**. Dendrogram showing the relatedness of 7 *S. *Enteritidis isolates using SPSS software program, HE1, HE2 and HE3: *S.* Enteritis isolates from human origin, FE1, FE2, FE3 and FE7: *S.* Enteritidis isolates from food origin

**Fig. 3:**
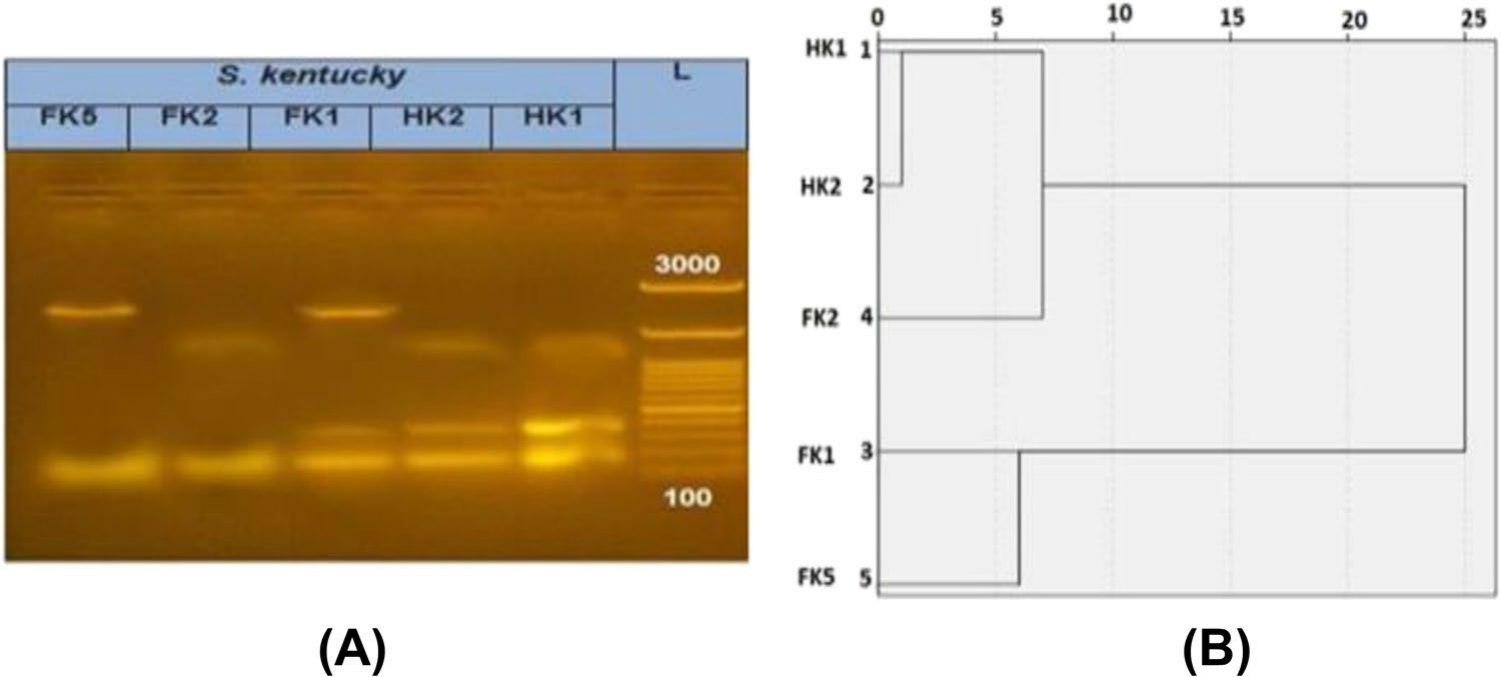
ERIC-PCR of S. Kentucky. **A**. ERIC-PCR finger printing of 5 *S.* Kentucky isolates in 1.5% agarose gel, L: 100bp molecular marker, FK1, FK2 and FK5: *S.* Kentucky isolates from food origin, HK1 and HK2: *S.* Kentucky isolates from human origin. **B**. Dendrogram showing the relatedness of 5 *S.* Kentucky isolates using SPSS software program, FK1, FK2 and FK5: *S.* Kentucky isolates from food origin, HK1 and HK2: *S*. Kentucky isolates from human origin

ERIC-PCR typing of 7 strains *S.* Enteritidis showed 5 ERIC-PCR type clusters. The maximum dissimilarity was 25%, and these in a common band between all strains. *S.* Enteritidis clusters in 2 main groups A and B. Group A have 2 human strain HE2 and HE3 and one food origin FE7 with a similarity 99% in its subdivision A1 and A2 composed of one food origin (F1). Group B divided into B1 (FE2) only and B2 (FE3) only with a similarity ratio ≥ 93%. ERIC-PCR analysis of 5 strains *S.* Kentucky showed 4 ERIC-PCR types, clustered in 2 main groups A and B with similarity 75%. Group A composed of HK1, HK2 human origin, and FK2 from food. The subdivided A1 composed only from HK1 and HK2 with a similarity 99%, while A2 composed only from FK2. The similarity group between A1 and A2 was 93%. Group B composed of FK1 in subgroup B1 and FK5 in subgroup B2 with a similarity ≥ ratio 94% as shown in Figs. 2 and 3.

## Characterization of silver nanoparticles (Ag-NPs by SEM and TEM)

**Fig. 4:**
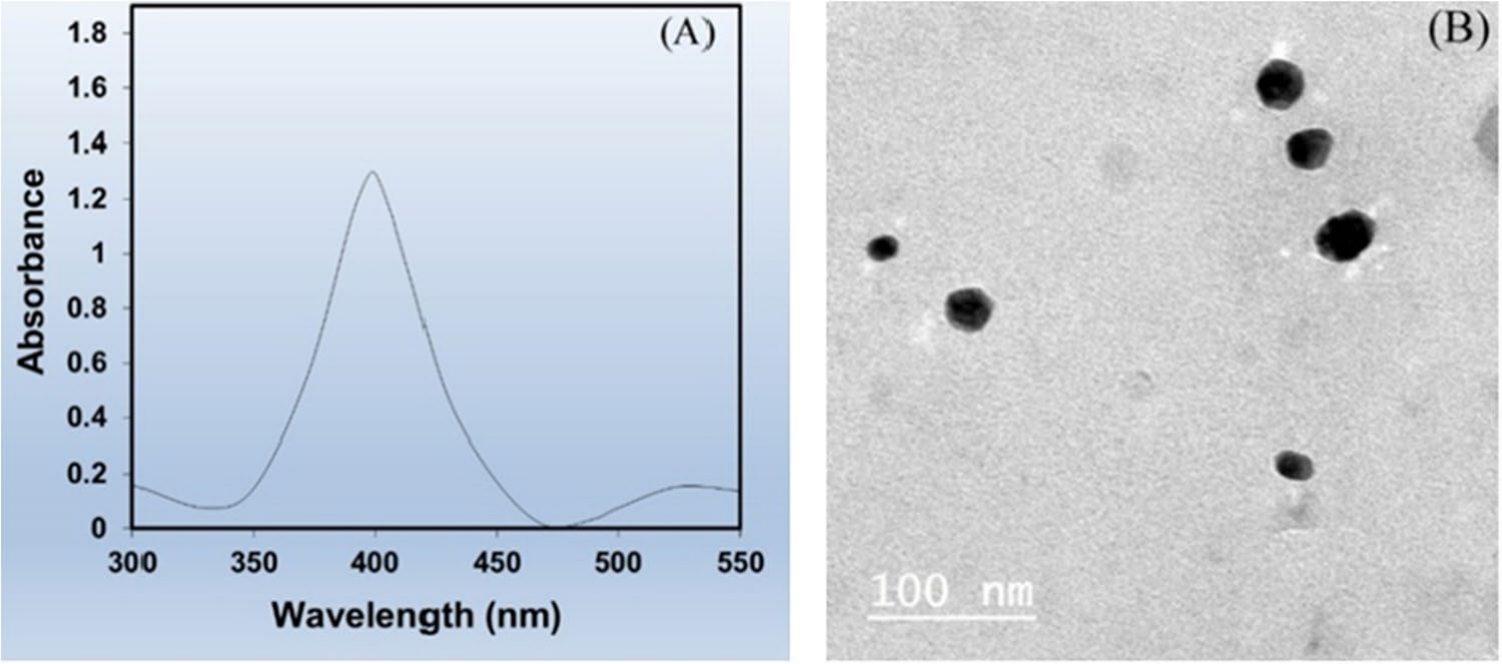
size and shape of Ag-NPs in solution by UV- Vis absorption spectra and TEM on TEM; JEOL-JEM-1230; high resolution. **A** Measurements of size distribution of (Ag NPs) by dynamic light scattering. **B** Transmission electron microscopy image of Ag NPs

**Fig. 5:**
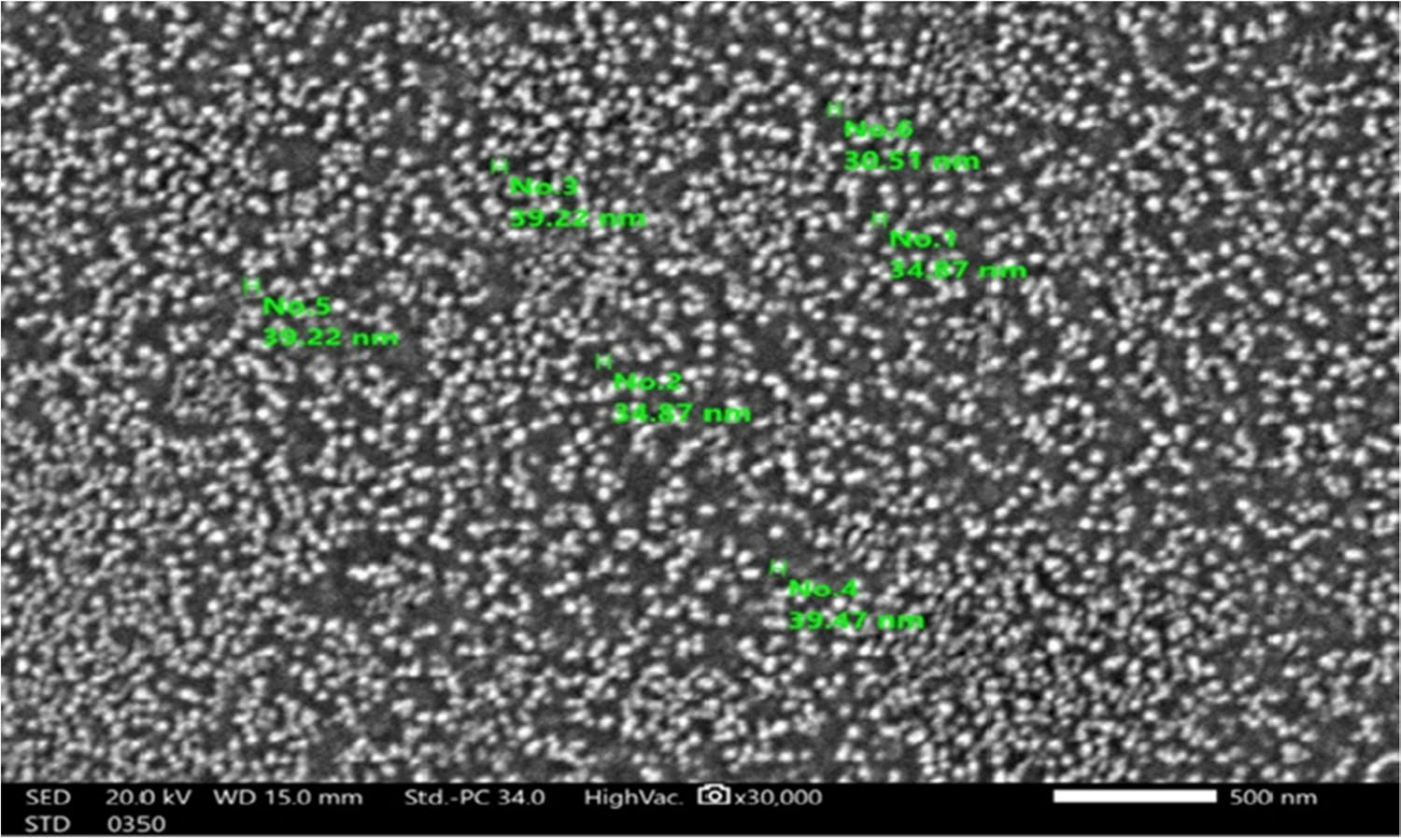
Size and shape of Ag NPs in solution by use SEM shown size 30–40 nm

Characterization of silver nanoparticles (Ag-NPs) prepared by chemical reduction from aqueous solution of silver nitrate using various analytical techniques as shown in Figs. 4 and 5.

## Results of Ag-NP effect (50 ppm) on S. Enteritidis cells revealed by SEM image after treatment for 3 h and 24 h

**Fig. 6:**
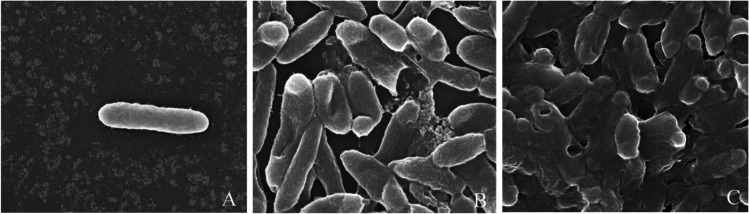
**A** Untreated cells *S. *Enteritidis showing intact cells. Cells had uniform electron density, straight with rounded end. **B** Treated *S. *Enteritidis cells with Ag-NP particles at concentration of 50 ppm after 3 h by SEM; we can notice morphological damage–disruption of cell wall. **C ***S. *Enteritidis cells after 24 h treatment with 50 ppm Ag NPs presented rupture in the cell wall and also losing shape of bacterial cell and complete bacterial lysis only some bacteria loose the rounded ends, the ends much as pointed and less extent in its width

The results of Ag-NP effects on *S.* Enteritidis bacterial cells revealed by SEM, *S.* Enteritidis cells with Ag-NP particles at concentration of 50 ppm after 3 h, we can notice morphological damage disruption of the cell wall, and can noted *S.* Enteritidis cells with Ag-NP particles at concentration of 50 ppm after 24 h Ag-NPs; the cell and complete bacterial lysis was observed at 24 h as shown in Fig. 6.

## The results of Ag-NP effects (50 ppm) on S. Kentucky cells revealed by SEM image, after treatment for 3 h and 24 h

**Fig. 7:**
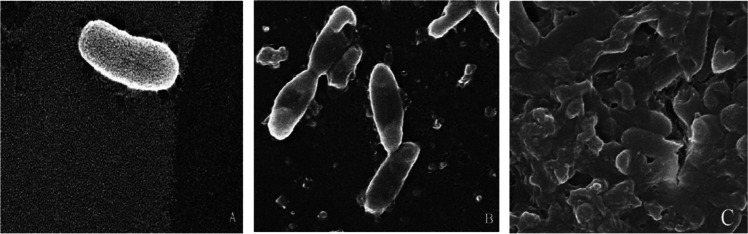
**A** Untreated cells *S. *Kentucky showing intact cells. Cells had uniform electron density, straight with rounded end. **B** Treated *S. *Kentucky cells with Ag-NP particles at concentration of 50 ppm after 3 h by SEM, we can notice morphological damage–disruption of cell wall, **C ***S. *Kentucky cells after 24 h treatment with 50 ppm Ag-NPs presented rupture in the cell wall and also, losing shape of bacterial cell and complete bacterial lysis. Some bacteria decreased in width and other are completely damaged, losing its rounded ends

The results of Ag-NP effect on *S.* Kentucky bacterial cells revealed by SEM, *S.* Kentucky cells with Ag-NP particles at concentration of 50 ppm after 3 h, we can notice morphological image disruption of the cell wall and can noted *S.* Kentucky cells with Ag-NP particles at concentration of 50 ppm after 24 h, the cells presented rupture in the cell wall, and also, losing shape of bacterial cell and complete bacterial lysis was observed at 24 h as shown in Fig. 7.

The preparing of silver nanoparticles (Ag-NPs) showed its characterization by UV–VIS spectroscopy, transmission electron microscope (TEM), and scanning electron microscope (SEM) average size 26.5 nm, 30–40 nm, and 45 ± 5 nm have the detected (MIC) minimum inhibitory concentration showed strong antibacterial activity. The results of disc diffusion method showed no significant difference due to different sizes, but the time of contact and concentration of Ag-NPs directly affected the bacterium as from the electron microscopy images.

We noticed the MIC and MBC of *S.* London half that of *S.* Kentucky as in previous study the same strain using Zn-NPs obtained by **(**Wang et al. 2018) method. Interestingly, MIC and MBC were also the same; i.e., MIC was 2.5 µg/ml and 5 µg/ml for *S.* London and *S.* Kentucky, respectively, and MBC was 5 µg/ml and 10 µg/ml for *S.* London and *S.* Kentucky, respectively.

SEM images show all the untreated control cells have intact and smooth surface. Both *Salmonella* spp. remained normally rod shaped, Ag-NPs adhered mainly to cell wall of *S.* Enteritidis, and some cells show penetration of Ag-NPs inside, thus modified the cell and characterized by the formation of “pits” allowed entry of Ag-NPs into the cell and may be death. At the site of Ag-NP adsorbed aggregation, there is widening of the periplasmic space in which Ag-NPs had accumulated the disrupted lysis cell membrane, damaged cytoplasm, and cell deformity.

In case of *S.* Kentucky Ag-NPs were able to damage the cell wall, but did not enter the cell, the cells appear to be shorter and more compact, suggesting there could be some leakage of the cellular content caused by the treatment; no lysis was noticed.

### In vivo

Two out of five chicks injected only *S.* Kentucky died before the end of the first week, i.e., mortality rate 2/5. This group showed re-isolation of *Salmonella* on XLD agar from positive slide agglutination test and from liver and spleen. The pathological lesion was severe hepatitis with multiple pale foci and pericarditis, peritonitis, airsacculitis, and pneumonia.

## Discussion

This is the primary nationwide survey for prevalence of *Salmonella enterica* serovar species isolated from calves, food, poultry, and human food in northern west provincial five cities Matrouh, Alexandria, Damanhour, Mahmoudia, and Desouq.

The serovar analysis has shown *S.* Enteritidis serovar as the dominant serovar and high-frequency *S.* Kentucky isolation. Recent reports from Brazil, Poland, Malaysia, China, and Greece have shown the same *S.* Enteritidis serovar with frequencies ranging from 34 to 86% which reveals coincident rise of the serovar around the world (Wen et al. 2017; Fernandes et al. 2006; Sadkowska-Todys and Czarkowski 2014; Spiliopoulou et al. 2007) and the high frequency of *S.* Kentucky was reported in Egypt (Osman et However, *Salmonella* species dominant serotypes have changed over time in various geographical locations (Fardsanei et al. 2016).

In antibiotic sensitivity testing approximately 3 from 4 strains of *S. enterica* were multidrug-resistant MDR, in which bacterial strains are resistant to more than three classes of antibiotics (Magiorakos et al. 2012), this was expected due to worldwide spreading of MDR bacteria (Hawkey et al. 2019; Coipan et al. 2020 and WHO 2021). As previously documented for clinical isolates, the variation between the five cities is related to reduce prescribing of particular drugs in certain cities (Gatto et al. 2006; Davin-Regli et al. 2008). The isolation of one strain of bacteria resistant to more than ten antibiotics in poultry has led us to fear that there will be no effective medicines available to treat resistant infections one day (Handayani et al., 2017). Chloramphenicol, which was blindly supplied by veterinarians in Egypt for diarrhoea, was found to stimulate the MDR response by activating the production of certain regulatory mRNA or other genes, according to Davin-Regli et al. (2008). While point mutations in DNA gyrase genes or activation of the efflux pump may cause enhanced resistance in food-borne isolates (Meakins et al. 2008), horizontal transfer and clonal expansion of resistance genes may occur among food-producing animals and humans (Hawkey 2008). The majority of developed countries utilize a regular surveillance and monitoring a system for antimicrobial drug resistance (AMP) that updated regularly to identify changes in antimicrobial resistance (WHO 2021), for example, national antimicrobial resistance monitoring system (NARMS) in the united states and the Danish integrated antimicrobial resistance monitoring and research program (DANMAP) in Denmark. The developing countries like Egypt did not have these systems (Vernet et al. 2014). Antibiotic resistance and serotyping methods (historically) were provided data to be used for short epidemiological studies, trends in well-defined geographical areas, and comparing between different countries (Tenover et al. 1997; Maccanell et al. 2013). Today, these methods in the increased MDR bacteria and time cost in my opinion do not have practical value as previously mentioned by Ranjbar et al. (2014). Recently, DNA-based typing method like ERIC-PCR subtype becomes indispensable to study the epidemiology of most microbial pathogens (Ranjbar et al. 2014). Our investigation used ERIC-PCR infer transmission of *Salmonella* Enteritidis from Mahmoudia city to Alexandria city as food born pathogen and cross transmission between calf and poultry in the same farm yards. Moreover, we reported 5 ERIC-PCR types of *S.* Enteritidis isolated from food and human. A previous study reported six ERIC types of *S.* Enteritidis isolated from food and patients from north Morocco (Ammari et al. 2009) and from southern Brazil revealed 3 ERIC types (Oliveira et al. 2007) and from India from diverse origin were categorized into clusters (Anjay et al. 2015) and in Iran into 4 clusters (Fardsanei et al. 2016) showed five different banding patterns with two major common types representing 76.6% of the 30 isolates they examined, each of which considered of both clinical and food isolates. The CT 3 only includes clinical, while CT4 included food samples.

For *S.* Kentucky, the 5 isolates (2 from human and 3 from food) were clustered in two main groups A and B subdivided into (AI, A and B1, B) giving 4 ERIC-PCR types with similarity 75%. The two human samples clustered in A1 with one food origin had 99% similarity, while that from food are in cluster A2. The similarity between A1 and A2 was 93%. B1 and B2 each composed of one from food origin with similarity ≥ 94%. ERIC-PCR results infer that all isolates which were phenotypically homogenous also genotypically homogenous were clonally dispersed among food, human population, animal, and poultry, and may continue to exist over considerable period of time on northern west Egypt and spread in different time occasion, supported the notion that infected animals, poultry, and humans are important source of contamination on the environment and food chain.

In vitro, the concentration of Ag-NPs and contact time directly affect the bacterial activity. As the increased of reactivity with decreasing particle size increased the number of attached cells due to large surface area, it provides better contact with microorganisms. The number in the different methods was used for preparing Ag-NP particles (Morones et al. 2005; Lok et al. 2006; Dror-Ehre et al. 2009; Kourmouli et al. 2018). Due to large surface area provide Ag-NPs, better contact with microorganism independent then size (Toker et al. 2013). The difference in method of synthesis provides different electrical charges that increase in the positive, less in neutral, and weak in negative (Abbaszadegan et al. 2015). This study showed that Ag-NPs of the exact same size and same synthesis method unyield vastly different MIC and MBC value simply on *Salmonella enterica* serovar species; time for interaction plays important role in damage and lysis in bacteria and initial concentration. The concentration and time of contact significantly affect the bacterial response. Kourmouli et al. (2018) concluded that the apparent antibacterial behavior is attributed to the ion Ag-NP release rather than to their unique size-dependent properties. Previously, the number of C.F.U. of *Salmonella* spp. was significantly reduced with increasing concentration (Raffi et al. 2008; Guzman et al. 2012; El- Sherif and Ali 2020) and the destruction and damage of cell wall lysis, expulsion of cellular content, mechanisms such as negative regulation of porins, chromosomal resistance genes, or plasmid with resistant genes have been proposed (Salas-Orozco et al. 2019). It has been implied by many authors that Ag-NPs are capable of attaching to bacterial cell membrane and as well as entry into cells (Pal et al. 2007; Dror- Ehre et al. 2009); they did not use the electron microscopy. Others reported that only Ag-NPs with diameter less than 10 nm were capable of entry *E. coli* and *Pseudomonas aeruginosa* (Morones et al. 2005), while 80 nm size can accumulate within after the addition of chloramphenicol (Xu et al. 2004). However, Samberg et al. (2011) showed ruptured and damaged bacteria with Ag-NP agglomerate nearby, but the electron microscopy image confirmed the actual penetration of Ag-NPs into whole bacteria. The success of Ag-NPs as effective antimicrobial is strongly strain dependent, since sensitivity to action of Ag-NPs, and MIC, thus is probably due to cell wall thickness differences. Berton et al. (2014), He et al. (2016), and Stoyanova et al. (2016) said that thus due to sensitivity, while Silver et al. (2006) is probably genetic factors specifically intrinsic of each strain including the presence of specific determinant of resistance, the possible mechanism of action is that the metal nanoparticles are carrying the positive charge and microbes the negative charges which create the electromagnetic attraction and microbe get oxidized (Rezaei Zarchi et al., 2010) or nanoparticles which react with thiol group (-SH) of the protein present in the cell surface of bacteria leads to lysis (Zhang 2013).

In vivo, in this work, silver nanoparticles (Ag-NPs) proved as proficient prevention and therapeutic agents due to their outstanding, physical mode of action (Meena et al. 2018). Many theories had been proposed the unclear blurred mechanism of action of Ag-NPs by which it exerts their antimicrobial effect, but two main hypotheses have been exposed: a direct interaction after adhesion on bacterium cell wall and the release of ionic silver (Gugala et al. 2022). Recently, reduction in the silver ions (Ag) concentration of polymer-coated Ag-NPs did not affect their antibacterial efficacy (Ashmore et al. 2018); mechanisms such as negative regulation porins, chromosomal resistance genes, or plasmid with resistance genes have been proposed (Salas-Orozco et al. 2019). Finally, in the twentieth century, a popular belief was that except for causing Angria, silver was relatively non-toxic to mammalian cells; however, studies conducted recently have shown that at the nanoscale, silver-based materials can exhibit significant toxicity to animals and human cells; these issues must be addressed before people rush to indulge into the nanosilver boom (Chen and Schluesener 2008). So, the silver nanoparticles could be used in the treatment in the intestinal tract of poultry (usual place of habitat and propagation of *Salmonella*), in this original research, a virulent MDR strain of *S.* Enteritidis fails to cause and continue its pathogenesis thus occur through combine Ag-NPs to *S.* Enteritidis cells in the intestinal tract of days old chicks broilers. Nokhodchi et al. (2012) in their review article clarify the challenge in drug delivery to combat *Salmonella* spp. and fail of new antibiotic to eradicate the pathogens completely, due to difficulty of transport of antibiotic retail through membrane (Drulis-Kawa and Dorotkiewicz-Jach 2010); reduced cell membrane permeability has been dedicated as a key mechanism of resistance to antibiotic (Davin-Regli et al. 2008), while nanoparticles adhere to cell membrane of *Salmonella* and in some species of *Salmonella* can release into the interior of the bacteria as in case of *Salmonella* Enteritidis, thus can interfere with bacterial resistance and infection mechanism which involve low membrane permeability or efflux system (Mugabe et al. 2006). But the dosage of silver nanoparticles differs according to its concentration (Ranjan et al. 2009). Generally, in the twentieth century, a popular belief was that except for causing Angria, silver was relatively nontoxic to mammalian cells. However, studies conducted in recent decades have shown that at the nanoscale, silver-based materials can exhibit significant toxicity to animal and human cells. These issues must be addressed before people rush to indulge into the nanosilver boom (Chen and Schluesener 2008); nowadays, nanoparticles have been used in disinfection textile fabrics, water disinfection, medicine and food packing, and preservation (García-Barrasa et al. 2011; Toker et al. 2013; Antonio et al. 2014; Mihindukulasuriya and lim 2014). We here report the results of the primary investigation for the prevalence of *Salmonella enterica* serovar spp. isolated from food, animals, poultry, and hospital patient in large five cities at Northern West Egypt. Results showed sources importance as vehicles for the dissemination of the *Salmonella* and posed a critical health risk for the populations. Electron microscopy effect on *S*. Enteritidis and *S*. Kentucky and antibiotic-resistant patterns of the isolated *Salmonella* were explored; therefore, close surveillance of antimicrobial resistance in bacteria should be established as a priority. Our data provide a base for further investigations.

## Data Availability

The datasets used and/or analyzed during the current study are available from corresponding author on reasonable request.

## Declarations

### Guidelines

All methods were carried out in accordance with relevant guidelines and regulations.

### ARRIVE guidelines

The authors confirm that the study was carried out in compliance with the ARRIVE guidelines.
